# Optimal Synthesis of Four-Bar Linkage Path Generation through Evolutionary Computation with a Novel Constraint Handling Technique

**DOI:** 10.1155/2018/5462563

**Published:** 2018-11-01

**Authors:** Suwin Sleesongsom, Sujin Bureerat

**Affiliations:** ^1^Department of Aeronautical Engineering, International Academy of Aviation Industry, King Mongkut's Institute of Technology Ladkrabang, Bangkok 10520, Thailand; ^2^Sustainable and Infrastructure Research and Development Center, Department of Mechanical Engineering, Faculty of Engineering, Khon Kaen University, Khon Kaen 40002, Thailand

## Abstract

This paper presents a novel constraint handling technique for optimum path generation of four-bar linkages using evolutionary algorithms (EAs). Usually, the design problem is assigned to minimize the error between desired and obtained coupler curves with penalty constraints. It is found that the currently used constraint handling technique is rather inefficient. In this work, we propose a new technique, termed a path repairing technique, to deal with the constraints for both input crank rotation and Grashof criterion. Three traditional path generation test problems are used to test the proposed technique. Metaheuristic algorithms, namely, artificial bee colony optimization (ABC), adaptive differential evolution with optional external archive (JADE), population-based incremental learning (PBIL), teaching-learning-based optimization (TLBO), real-code ant colony optimization (ACOR), a grey wolf optimizer (GWO), and a sine cosine algorithm (SCA), are applied for finding the optimum solutions. The results show that new technique is a superior constraint handling technique while TLBO is the best method for synthesizing four-bar linkages.

## 1. Introduction

Since the last decade, many researchers have tried to solve the optimization for path generation of four-bar linkages using metaheuristic (MH) algorithms. The objective of path generation problem is to find dimensions of a mechanism, which minimize the target path and the actual path of a point on the coupler link. Path synthesis is one type of kinematic syntheses of four-bar mechanisms [1–13] in which such syntheses are basically classified into two groups. The first category is called dimensional synthesis [1, 2, 4, 5, 7–10]. This synthesis type aimed to find significant link lengths to achieve desirable function, path, and motion generation. The second synthesis is called type synthesis [6, 11] where a designer initially specifies a predefined motion transmission and is supposed not initially to know the mechanism type. This method is analogous to topology design in structural optimization. Having finished synthesizing, a certain mechanism type is received. Position analysis of the four-bar mechanism can be categorized into two groups. The first one is a vector loop or loop closure equation, which is the most traditional method in kinematic analysis, and it is one of the most popular analyses for path synthesis [1, 2, 4, 5, 7–15]. This equation can be solved by using Freudenstein equation. The second analysis technique is a straight forward and a simple method for position analysis involving the use of trigonometric laws for triangles, e.g., the law of cosine [3, 16, 17], whereas the six-bar linkage for steering mechanism also uses the same technique [18]. This work proposes a new computing technique for four-bar linkage position analysis by employing the concept of drawing an arbitrary rectangle using two circles.

The mechanism synthesis can be converted into optimization problem and be solved by using optimizers, where both nongradient- and gradient-based algorithms have been solved this problem. Recently, a nongradient-based optimizer, e.g., evolutionary algorithms (EAs) or metaheuristics (MHs), is a more popular selection in solving such optimization problems. It has been found that the advantages of using MHs are robustness, simplicity of use, and independence of function derivatives; however, they unavoidably lack convergence speed and consistency. At present, many algorithms in this group have been developed, which are expected to enhance in both convergence speed and consistency. Some of the most frequently used MHs for path synthesis are differential evolution (DE) [2, 3, 5, 8, 9, 11, 13], genetic algorithms (GA) [5, 13], particle swarm optimization (PSO) [5, 13], and an imperialist competitive algorithm (ICA) [13], etc. The use of gradient-based method, on the other hand, is somewhat questionable to deal with global optimization and nonsmooth constraints in the path synthesis. Nevertheless, if those aforementioned factors can be alleviated, the advantages of the gradient-based method are better convergence rate and consistency. In the literature, many researchers have combined MHs and a gradient-based optimizer for solving many kinds of real world problems, which is called a hybrid algorithm. Especially for part synthesis, the hybrid optimizers are introduced as the ant gradient [1], hybrid GA [4], and hybrid GA with sequential quadratic programming (SQP) [12]. The hybridization between two or more MHs was also studied such as hybrid GA-DE [7]. Furthermore, the path synthesis is extended to multiobjective optimization, which was solved by using a multiobjective genetic algorithm (MOGA) [10]. From the review literature, it was found that some MHs have been used for solving this task except the work by Sleesongsom and Bureerat [17]; therefore, one of the objectives of this paper is to present the comparative performance of a number of currently used MHs. Those algorithms include the artificial bee colony optimization (ABC), adaptive differential evolution with optional external archive (JADE), population-based incremental learning (PBIL), teaching-learning-based optimization (TLBO), the real-code ant colony optimization (ACOR), a grey wolf optimizer (GWO), a Jaya algorithm (Jaya), and a sine cosine algorithm (SCA).

The path generation is a mechanism synthesis to make a point on a coupler link move along the target path; thus, the objective function is the minimization of the sum square error between the target path and the actual path [5]. The design problem is a constrained optimization problem that comprises two constraints. The first constraint is set for the shortest link in the mechanism to be able to rotate with a complete revolution (crank) in either direction (clockwise or counter clockwise). The second constraint is assigned such that the four link lengths satisfy the Grashof criterion which results in a crank-rocker. From previous work, a simple exterior penalty function technique had used to deal with these constraints [1–5, 7, 8, 10–13]. The new technique proposed by [17] to neglect the first constraint from the optimization problem, which found new technique, provided the better result than the traditional exterior penalty technique. Additionally, the technique had improved in the result, but it increased in time consuming. From the present study can be concluded that the constraint handling technique is an inefficient technique, which needs an improvement [9, 13, 14]. Phukaokaew et al. [14] studied the number of unsuccessful runs from performing MHs for 30 runs, where the path synthesis optimization problems employ the penalty function (PF) technique. This means there is no guarantee that using this technique can promote the good results. The reason is that using the penalty technique leads to an overly narrow feasible region. As a consequence, MHs, which mostly have slow convergence rates, struggle to reach an optimum. Ebrahimi and Payvandy proposed the way to improve the constraint handling technique, which was still based on a penalty function, and they believe the proposed method can enhance the search performance [13].

This paper focuses on two aspects of investigation. Firstly, a new constraint handling technique for path synthesis of a four-bar linkage using MHs is proposed. The method is based on repairing illegitimate design solutions to become feasible solutions. The second investigation is to study the performance comparison of a number of established MHs for solving four-bar linkage path synthesis with the new constraint handling technique, where both convergence rate and consistency of the methods are measured.

The rest of this paper is organized as follows.  Section 2 proposes an alternative position analysis of a four-bar linkage. The optimization problem and the constraint handling technique are detailed in Section 3. The numerical experiments are given in Section 4, while the design results are discussed in Section 5. The discussions and conclusions of the study are finally drawn in Sections 6 and 7, respectively.

## 2. Position Analysis of Four-Bar Linkages

**Figure 1:**
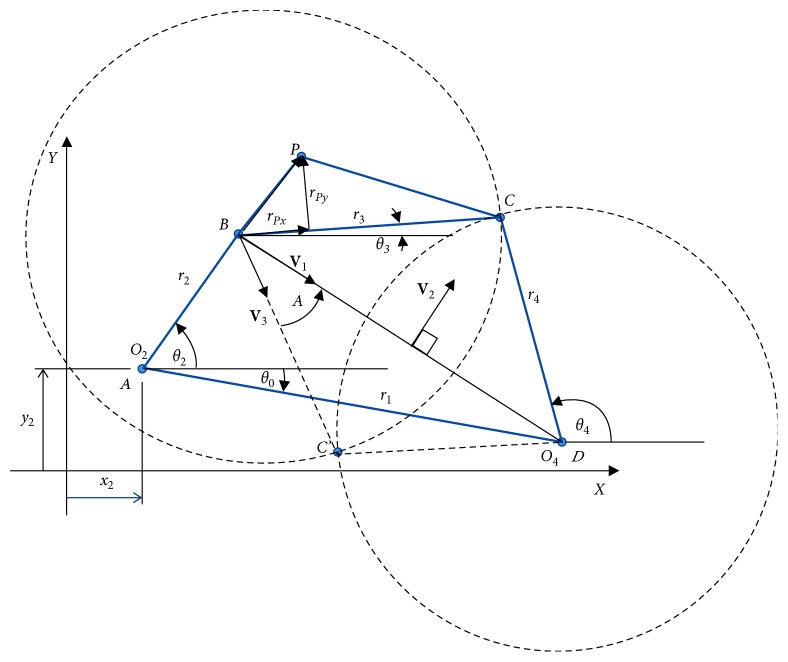
Four-bar linkage in a global coordinate system.

The kinematic diagram of a four-bar linkage is shown in Figure 1. The four-bar linkage is the simplest and most commonly used linkage in many engineering applications. It is composed of a kinematic chain of four binary links connected with four revolute joints (denoted by capital letters) with one link being assigned as a frame. Applications for this mechanism are a window wiper, a door closing mechanism, rock crushers, etc. [9]. Based on the Gruebler equation for planar mechanisms, the mobility or degree-of-freedom of the mechanism is one; thus, it is a constrained mechanism fully operated by one actuator. The path generation for a four-bar linkage is a dimension-based design of the four-bar linkage lengths (*r*_1_, *r*_2_, *r*_3_, *r*_4_) and other parameters, which makes the trace point (*x*_*P*_, *y*_*P*_) on the coupler link follow the desire path (*x*_*d*_, *y*_*d*_).

Let the coordinates of the joints *O*_2_ and *O*_4_ be (*x*_2_, *y*_2_) and (*x*_4_, *y*_4_), respectively, so the coordinates of points *B* and *O*_4_ can be computed as

(1) $$\begin{array}{c} x_{B}=x_{2}+r_{2}\;\;\cos\left(\theta_{2}\right),\\ y_{B}=y_{2}+r_{2}\;\;\sin\left(\theta_{2}\right),\\ x_{4}=x_{2}+r_{1}\;\;\cos\left(\theta_{0}\right),\\ y_{4}=y_{2}+r_{1}\;\;\sin\left(\theta_{0}\right), \end{array}$$

where the angular positions *θ*_0_ and *θ*_2_ are positive counter-clockwise. The positions of points *C* and *C*′ are the intersection points of two circles as illustrated in Figure 1, which is calculated by a vector approach. First, let *B* and *O*_4_ be the centres of the circles with radii *r*_3_ and *r*_4_, respectively, and let *d* be the distance between *B* and *O*_4_. Then, generate **V**_1_, the unit vector from *B* to *O*_4_, and **V**_2_, the unit vector perpendicular to **V**_1_. Given that **V**_3_ is the vector from *B* to one of the intersection points, the angle between **V**_1_ and **V**_3_ denoted by *A* is solved using the law of cosines

(2) $$\begin{array}{c} r_{3}^{2}=r_{4}^{2}-d^{2}+2r_{3}d\;\;\cos\left(A\right). \end{array}$$

The intersection points can then be obtained as

(3.1) $$\begin{array}{c} r_{C}=r_{B}+\left(r_{3}\;\;\cos\;\;A\right)V_{1}+\left(r_{3}\;\;\sin\;\;A\right)V_{2}, \end{array}$$

(3.2) $$\begin{array}{c} r_{C^{\prime }}=r_{B}+\left(r_{3}\;\;\cos\;\;A\right)V_{1}-\left(r_{3}\;\;\sin\;\;A\right)V_{2}. \end{array}$$

The coupler curve is formed when the crank link rotates. From Figure 1, the coupler point coordinates (**r**_*P*_) in the global coordinate can be expressed as

(4) $$\begin{array}{c} r_{P}=r_{B}+r_{P/B}=r_{B}+r_{Px}+r_{Py}=r_{B}+r_{Px}e_{Px}+r_{Py}e_{Py}, \end{array}$$

where **e**_*Px*_ is a unit vector in the direction from *B* to *C* and **e**_*Py*_ is a unit vector perpendicular to **e**_*Px*_. The distances **r**_*Px*_ and **r**_*Py*_ can be computed using the given dimensions of *r*_3_, *PB*, and *PC*. Also, they can be set as design variables for optimal path synthesis. These vector forms of position analysis are used for four-bar linkage synthesis in this paper.

## 3. Optimization Problem and Constraint Handling

### 3.1. Optimization Problem

The path synthesis problem is converted to an optimization problem with an objective function expressed as the sum of square errors between the distances of **r**_*d*_ and their corresponding **r**_*P*_. A set of the input angles (*θ*_2_) is assigned as design variables if the prescribed timing is not given. There are two major constraints. The first constraint is set in such a way that the generated mechanism type is a crank-rocker. This leads to two constraints: (i) sum of the shortest and longest links of linkage must be less than the sum of two remaining links and (ii) the shortest link must be an input link where one of its nearby links is set as a frame. The constraint (i) is denoted with Equation (7), while the constraint (ii) is denoted with Equation (6). In cases where the prescribed timing is not promoted, the second constraint is that the input values of *θ*_2_ must be in either ascending or descending order. The constraint (ii) is added to the design problem, which is denoted by *θ*_2_^*i*^, where *i*=1. The optimization problem can then be written as

(5) $$\begin{array}{c} \min\;f_{\text{obj}}\left(x\right)=\overset{N}{\underset{i=1}{\sum }}\left[{\left(x_{d,i}-x_{P,i}\right)}^{2}+{\left(y_{d,i}-y_{P,i}\right)}^{2}\right], \end{array}$$

subject to

(6) $$\begin{array}{c} \min\left(r_{1},r_{2},r_{3},r_{4}\right)=\text{crank}, \end{array}$$

(7) $$\begin{array}{c} 2\min\left(r_{1},r_{2},r_{3},r_{4}\right)+2\max\left(r_{1},r_{2},r_{3},r_{4}\right)<\left(r_{1}+r_{2}+r_{3}+r_{4}\right), \end{array}$$

(8) $$\begin{array}{c} \theta_{2}^{i}<\theta_{2}^{i+1}<\cdots <\theta_{2}^{N},\;i=1, \end{array}$$

(9) $$\begin{array}{c} x_{l}\leq x\leq x_{u}, \end{array}$$

where **x**={*r*_1_, *r*_2_, *r*_3_, *r*_4_, **r**_*Px*_, **r**_*Py*_, *θ*_0_, *x*_*O*_2__, *y*_*O*_2__, *θ*_2_^*i*^}^*T*^, *N* is the number of points on prescribed or target curve, and *θ*_2_^*i*^ are input link angles. **x**_*l*_ and **x**_*u*_ are lower and upper bounds of a design vector **x**, respectively.

A function evaluation is carried out for the optimization problem (5) by implementing the position analysis detailed in Section 2. To enable the position analysis, the constraints (6)–(8) must be first satisfied. In the previous studies, if the conditions are not met, the objective function will be modified by adding to it a great penalty value. However, in this work, the proposed path repairing algorithm (PRA) is assigned to repair all design solutions to always be feasible before performing position analysis of a four-bar linkage.

### 3.2. Constraint Handling

Normally, a traditional exterior penalty function technique has been used with the constrained optimization problem (5) [1–5, 7, 8, 10–13], but it was found to be inefficient [9, 13, 14, 17]. Using such a technique is questionable when prespecifying a penalty parameter. If the parameter is too small, the resulting optimum solution may be infeasible, but if it is too large, MH may not be able to find the optimum. This leads us to propose a new strategy to deal with the constraints called the path repairing technique. The technique can be separated into two parts as repair of the input link angle constraint (8) and the Grashof criterion constraint Equations (6) and (7).

#### 3.2.1. Input Link Angle Constraint

For an optimization without prescribed timing, a repairing technique for this phase is shown in Algorithm 1 where the input variables are **x**={*r*_1_, *r*_2_, *r*_3_, *r*_4_, **r**_*Px*_, **r**_*Py*_, *θ*_0_, *x*_*O*_2__, *y*_*O*_2__, *θ*_2_^*i*^}^*T*^. Once it is found that the values of *θ*_2_^*i*^ are not in either ascending or descending orders, the design variables **x** in the part of *θ*_2_^*i*^ will be repaired. Firstly, *N* − 1 uniform random number *α*_*i*_ ∈ [0.0001, 1] for *i*=1,…, *N* − 1 is generated. The lower bound is set to be 0.0001 in order to avoid repeated values of *θ*_2_^*i*^ in cases that some random numbers *α*_*i*_ become zeros. Those random values are then scaled in step 2 so that the sum of them does not exceed 2*π*. The first angular position of an input link is *θ*_2_^1^, its original value. Then, the next value of *α*_*i*_ for *i*=1,…, *N* − 1 is accumulatively added to the next input angle until the last value is obtained as *θ*_2_^*N*^=*θ*_2_^1^+*α*_1_+⋯+*α*_*N*−1_. Then, the new set of input angles is in an ascending direction before returning to the position analysis of a four-bar linkage. This concept was successfully used in a sprayed plate fin heat sink design [19]. The obtained sequence of input angles always obeys the constraint (8).

The vector of design variables for path synthesis of a particular four-bar linkage is **x**={*r*_1_, *r*_2_, *r*_3_, *r*_4_, **r**_*Px*_, **r**_*Py*_, *θ*_0_, *x*_2_, *y*_2_, *θ*_2_^1^, *θ*_2_^2^, *θ*_2_^3^, *θ*_2_^4^, *θ*_2_^5^, *θ*_2_^6^}^*T*^. Variables *r*_1_–*r*_4_ ∈ [5,60] are the link lengths of the linkage, and *θ*_2_^1^–*θ*_2_^6^ ∈ [0,2*π*] are the angular positions of link *r*_2_, also known as timings of the crank, while other variables are shown in Figure 1. The legitimated set of the timings must obey the condition *θ*_2_^1^ < *θ*_2_^2^ < *θ*_2_^3^ < *θ*_2_^4^ < *θ*_2_^5^ < *θ*_2_^6^. During an optimization process, if the set of timings is decoded as, for example, *θ*_2_^1^ = 0.5, *θ*_2_^2^ = 0.45, *θ*_2_^3^ = 0.67, *θ*_2_^4^ = 1.35, *θ*_2_^5^ = 4.50, and *θ*_2_^6^ = 2.10, they violate constraint (8). Then, Algorithm 1 is activated to repair these values. Five (for 6 timings) uniform random numbers are generated, for example, *α*_1_=0.5, *α*_2_=0.15, *α*_3_=0.75, *α*_4_=0.45, and *α*_5_=0.30. The values of *α*_*i*_ are then scaled down according to step 2 in Algorithm 1 to meet the condition *θ*_2_^1^–*θ*_2_^6^ ∈ [0,2*π*] leading to

(10) $$\begin{array}{c} \alpha_{1}=1.99\pi \times \frac{0.5}{\left(6-1\right)}=0.6252,\\ \alpha_{2}=1.99\pi \times \frac{0.15}{\left(6-1\right)}=0.1876,\\ \alpha_{3}=1.99\pi \times \frac{0.75}{\left(6-1\right)}=0.9378,\\ \alpha_{4}=1.99\pi \times \frac{0.45}{\left(6-1\right)}=0.5627,\\ \alpha_{5}=1.99\pi \times \frac{0.3}{\left(6-1\right)}=0.3751. \end{array}$$

The output modified values of *θ*_2_^1^–*θ*_2_^6^ are then computed as

(11) $$\begin{array}{c} \theta_{2}^{1}=\theta_{2}^{1}=0.5,\\ \theta_{2}^{2}=\theta_{2}^{1}+\alpha_{1}=1.1252,\\ \theta_{2}^{3}=\theta_{2}^{1}+\alpha_{1}+\alpha_{2}=1.3128,\\ \theta_{2}^{4}=\theta_{2}^{1}+\alpha_{1}+\alpha_{2}+\alpha_{3}=2.2506,\\ \theta_{2}^{5}=\theta_{2}^{1}+\alpha_{1}+\alpha_{2}+\alpha_{3}+\alpha_{4}=2.8133,\\ \theta_{2}^{6}=\theta_{2}^{1}+\alpha_{1}+\alpha_{2}+\alpha_{3}+\alpha_{4}+\alpha_{5}=3.1884. \end{array}$$

As a result, by using Algorithm 1, the timings are always feasible. The difference between *θ*_2_^6^ and *θ*_2_^1^ will never exceed 2*π*.

#### 3.2.2. Grashof's Criterion Constraint

With the same reasons as for the previous constraint, the dimensions of {*r*_1_, *r*_2_, *r*_3_, *r*_4_} must obey the conditions (6) and (7) so that the resulting mechanism is usable. Let the bound constraints of {*r*_*i*_} in Equation (9) be separately defined as

(12) $$\begin{array}{c} \left\{r_{1,l},r_{2,l},r_{3,l},r_{4,l}\right\}\leq \left\{r_{1},r_{2},r_{3},r_{4}\right\}\leq \left\{r_{1,u},r_{2,u},r_{3,u},r_{4,u}\right\}. \end{array}$$

Then, the relation can be found:

(13) $$\begin{array}{c} r_{\min}=\max\left\{r_{1,l},r_{2,l},r_{3,l},r_{4,l}\right\}\leq \left\{r_{1},r_{2},r_{3},r_{4}\right\}\leq \min\left\{r_{1,u},r_{2,u},r_{3,u},r_{4,u}\right\}=r_{\max}. \end{array}$$

The Grashof criterion repairing technique is shown in Algorithm 2. Firstly, four uniform random numbers {*δ*_1_, *δ*_2_, *δ*_3_, *δ*_4_} in the range of [0.0001, 1] are generated. The lower bound is set to be 0.0001 for the same reason as Algorithm 1. Then the auxiliary variables *S*_*i*_ for *i*=1,…, 4 are computed as

(14) $$\begin{array}{c} S_{2}=\delta_{2},\\ S_{3}=\delta_{2}+\delta_{3},\\ S_{4}=\delta_{2}+\delta_{3}+\delta_{4},\\ S_{1}=\delta_{1}+\delta_{2}+\delta_{3}+\delta_{4}. \end{array}$$

With this computation, it is concluded that *S*_2_ is their minimum and *S*_1_ is their maximum. These four values fulfil the Grashof criterion if *S*_2_ is an input crank. Condition (7) also holds if

(15) $$\begin{array}{c} S_{1}+S_{2}<S_{3}+S_{4},\\ \delta_{1}+2\delta_{2}+\delta_{3}+\delta_{4}<2\delta_{2}+2\delta_{3}+\delta_{4},\\ \text{or}\;\;\;\;\delta_{1}<\delta_{3}. \end{array}$$

Therefore, the computational steps 3-4 in Algorithm 2 are applied. Then, the values of {*S*_*i*_} are all scaled down so that max(*S*_*i*_)=*S*_1_ ≤ 1. The values of *b*_*i*_ can then be computed as

(16) $$\begin{array}{c} b_{i}=r_{\min}+\left(r_{\max}-r_{\min}\right)S_{i,}\;\text{for}\;\;i=1,\ldots ,4. \end{array}$$

Then, set *r*_2_=*b*_2_. The values of *b*_1_,   *b*_3_,   and *b*_4_ are assigned to be the lengths of *r*_1_,   *r*_3_ and *r*_4_, respectively. With such a computing scheme, the values of {*r*_1_, *r*_2_, *r*_3_, *r*_4_} returned from activating Algorithm 2 will always be feasible. In the search process of MH, when a function evaluation is revoked, feasibility of a design solution **x** will be checked. If it is infeasible, Algorithms 1 and 2 will be used to repair or alter the values of *θ*_2_^*i*^ and *r*_*i*_ in **x** and send them back to the main process of a metaheuristic. That means all design solutions in a population of MH are always feasible.

Given that, for example, *r*_1_=15, *r*_2_=10, *r*_3_=30, and *r*_4_=20, the Grashof criterion is not fulfilled since *r*_2_+*r*_3_ > *r*_1_+*r*_4_. These values will then be regenerated by using Algorithm 2. From step 1, given that the values of *δ*_1_–*δ*_4_ are randomly generated as *δ*_1_=0.01, *δ*_2_=0.55, *δ*_3_=0.25, and *δ*_4_=0.45, then the auxiliary variables are computed based on (14) as

(17) $$\begin{array}{c} S_{1}=0.01+0.55+0.25+0.45=1.26,\\ S_{2}=0.55=0.55,\\ S_{3}=0.55+0.25=0.80,\\ S_{4}=0.55+0.25+0.45=1.25. \end{array}$$

Since *S*_1_ is greater than 1.00, the values of the auxiliary variables are modified as

(18) $$\begin{array}{c} S_{1}=\frac{1.26}{1.26}=1.0000,\\ S_{2}=\frac{0.55}{1.26}=0.4365,\\ S_{2}=\frac{0.80}{1.26}=0.6349,\\ S_{4}=\frac{1.25}{1.26}=0.9921. \end{array}$$

Thus, the new link length values are obtained using (16):

(19) $$\begin{array}{c} r_{1}=5+\left(60-5\right)\times 1.0000=60.0000,\\ r_{2}=5+\left(60-5\right)\times 0.4365=29.0079,\\ r_{3}=5+\left(60-5\right)\times 0.6349=39.9195,\\ r_{4}=5+\left(60-5\right)\times 0.9921=59.5655, \end{array}$$

which results in a crank-rocker four-bar linkage. In cases where the generated value of *δ*_1_ is greater than that of *δ*_3_, their values are swapped. If they are equal, Step 3 in Algorithm 2 is activated ensuring that a crack-rocker is obtained after the repairing process.

## 4. Numerical Experiment

For evaluating the performance of the proposed path repairing technique, three path synthesis test problems of a four-bar linkage are used, whereas the optimizers are 7 established MHs. To validate the new approach, it will be compared with the exterior penalty function technique, which is traditionally applied in previous work. The path generation problems are detailed as [5, 14]:

**Case 1 .**

Path generation without prescribed timing

Design variables are

(20) $$\begin{array}{c} x={\left\{r_{1},r_{2},r_{3},r_{4},r_{Px},r_{Py},\theta_{0},x_{2},y_{2},\theta_{2}^{1},\theta_{2}^{2},\theta_{2}^{3},\theta_{2}^{4},\theta_{2}^{5},\theta_{2}^{6}\right\}}^{T}. \end{array}$$

Target points are *r*_*d*_^*i*^={(20,20), (20,25), (20,30), (20, 35), (20,40), (20,45)}.

Limits of the design variables are as follows:

(21) $$\begin{array}{c} 5\leq r_{1},r_{2},r_{3},r_{4}\leq 60,\\ -60\leq r_{Px},r_{Py},x_{2},y_{2}\leq 60,\\ 0\leq \theta_{0},\theta_{2}^{1},\theta_{2}^{2},\theta_{2}^{3},\theta_{2}^{4},\theta_{2}^{5},\theta_{2}^{6}\leq 2\pi . \end{array}$$

**Case 2 .**

Path generation with prescribed timing

Design variables are

(22) $$\begin{array}{c} x={\left\{r_{1},r_{2},r_{3},r_{4},r_{Px},r_{Py},\theta_{0},x_{2},y_{2}\right\}}^{T},\\ \theta_{2}^{i}=\left[\frac{\pi }{6},\frac{\pi }{3},\frac{\pi }{2},\frac{2\pi }{3},\frac{5\pi }{6},\pi \right]. \end{array}$$

Target points are *r*_*d*_^*i*^={(0,0), (1.9098, 5.8779), (6.9098, 9.5106), (13.09, 9.5106), (18.09, 5.8779), (20,0)}.

Limits of the design variables are

(23) $$\begin{array}{c} 5\leq r_{1},r_{2},r_{3},r_{4}\leq 50,\\ -50\leq r_{Px},r_{Py},x_{2},y_{2}\leq 50,\\ 0\leq \theta_{0}\leq 2\pi . \end{array}$$

**Case 3 .**

Path generation without prescribed timing

Design variables are

(24) $$\begin{array}{c} x={\left\{r_{1},r_{2},r_{3},r_{4},r_{Px},r_{Py},\theta_{0},x_{2},y_{2},\theta_{2}^{1},\theta_{2}^{2},\theta_{2}^{3},\theta_{2}^{4},\theta_{2}^{5},\theta_{2}^{6},\theta_{2}^{7},\theta_{2}^{8},\theta_{2}^{9},\theta_{2}^{10}\right\}}^{T}. \end{array}$$

Target points are *r*_*d*_^*i*^={(20,10), (17.66, 15.142), (11.736, 17.878), (5,16.928), (0.60307, 12.736), (0.60307, 7.2638), (5, 3.0718), (11.736, 2.1215), (17.66, 4.8577), (20,10)}.

Limits of the design variables are

(25) $$\begin{array}{c} 5\leq r_{1},r_{2},r_{3},r_{4}\leq 80,\\ -80\leq r_{Px},r_{Py},x_{2},y_{2}\leq 80,\\ 0\leq \theta_{0},\theta_{2}^{1},\theta_{2}^{2},\theta_{2}^{3},\theta_{2}^{4},\theta_{2}^{5},\theta_{2}^{6},\theta_{2}^{7},\theta_{2}^{8},\theta_{2}^{9},\theta_{2}^{10}\leq 2\pi . \end{array}$$

The first synthesis problem has a straight line target path without a prescribed timing. The second test problem has a circular prescribed path with prescribed timing while the third test problem has an elliptic path without prescribed timing. The optimizers used to tackle the test problems are 7 well-known and newly developed metaheuristics. Their optimization parameter settings are given below. The population size *n*_*P*_ = 100 is used for all algorithms, unless otherwise specified. It should be noted that the terms and variable definitions are from their original sources.

1. Artificial bee colony algorithm (ABC) [20]: the number of food sources for employed bees is set to be *n*_*P*_/2. A trial counter to discard a food source is 100.
2. Real-code ant colony optimization (ACOR) [21]: the parameter settings are *q* = 0.2 and *ξ* = 1.
3. Teaching-learning-based optimization (TLBO) [22]: parameter settings are not required.
4. Adaptive differential evolution with optional external archive (JADE) [23]: The parameters are self-adapted during an optimization process.
5. Population-based incremental learning (PBIL) [24]. The learning rate, mutation shift, and mutation rate are set as 0.5, 0.7, and 0.2, respectively.
6. Grey wolf optimizer (GWO) [25]: the GWO has only two main parameters to be adjusted, *a* and *C*, where *a* is decreased from 2 to 0 and *C* is randomly generated in the range of [0, 2].
7. Sine cosine algorithm (SCA) [26]. The parameter *r*_1_ decreases linearly from *a* = 2 to 0.

It should be noted that the parameter settings used in this paper are either the default values provided or suggested by their authors. Also, they have been used in some previous studies with acceptable results [17, 27–30].

The total number of iterations or generations for each optimization run is set to be 500. Any MH that uses a different population size will be terminated with the total number of function evaluations as 100 × 500. Each MH runs 30 times, so that to measure its convergence rate and consistency. It should be noted that most works in the literature tended to ignore examining the search consistency of MHs. This can be carried out by running MHs many times. In this paper, each MH is run 30 times for each optimization problem. The means of the objective function values obtained from the various MHs are used as their performance indicator. Also, the comparison based on nonparametric statistical Friedman test [17, 31] is employed. Thus, this study would be a proper baseline for the topic of using MHs for four-bar linkage path synthesis in the future.

In conclusion, the path repairing technique is proposed to increase the performance in solving four-bar linkage path synthesis. To evaluate the performance of the proposed technique, three path synthesis test problems and 7 established MHs are used to study. To validate the new constraint handling approach, it is compared with the results that obtained from using the classical exterior penalty function technique, the most popularly used technique for path synthesis. Moreover, statistical results including mean, minimum, maximum, and standard deviation are also reported.

## 5. Design Results

The optimization of path generation of four-bar mechanisms is to find the optimized link lengths and some other parameters, which minimize the objective function. Three case studies with and without prescribed timing are considered for performance testing of the optimizers and the new constraint handling technique. The mean values of objective functions are used as the main performance indicator, where the lower mean value is more reliable optimizer. The mean objective function value determines MH reliability as MH with lower mean value is likely to be more successful in solving the problem, even with only one optimization run.

**Figure 2:**
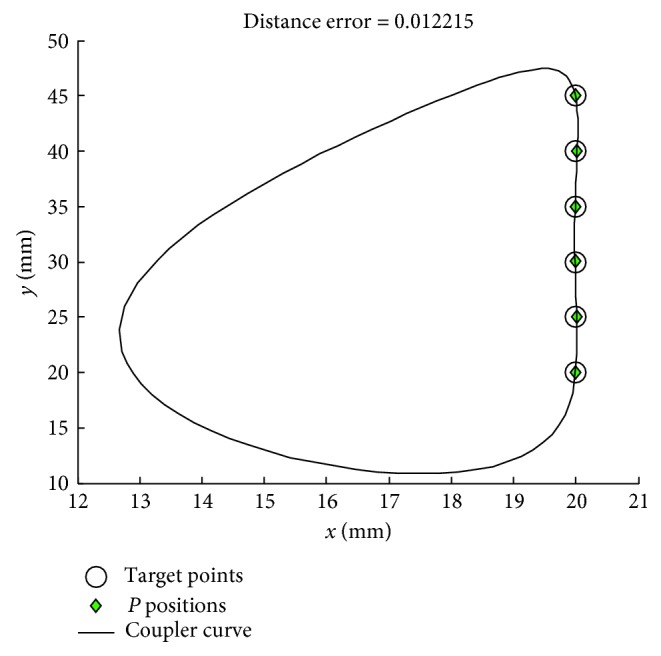
Best coupler curve obtained in Case 1.

**Figure 3:**
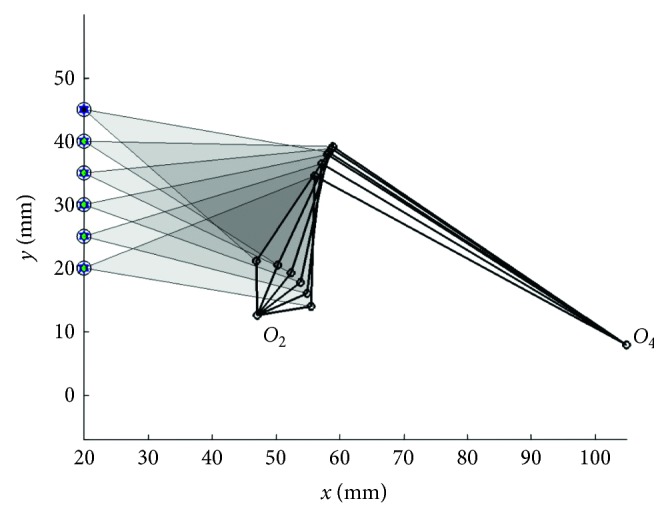
Best mechanism obtained in Case 1.

The results of Case 1 obtained from the seven optimizers with the novel path repairing technique and the penalty technique are shown in Tables 1 and 2 respectively. In the tables, the number of successful runs (a run that obtains a feasible solution), the mean objective function values from 30 optimization runs (Mean), the worse result (Max), the best result (Min), the standard deviation (Std), and the best linkage that gives the minimum objective function value of each algorithm are given. Each parameter computes based on the objective function defined as the sum of squares of the distances between the desired points, and the actual points, while the term “Error” in the tables is an average distance error of the best found solution computed as

(26) $$\begin{array}{c} \text{Error}=\frac{1}{N}\overset{N}{\underset{i=1}{\sum }}\sqrt{{\left(x_{d,i}-x_{P,i}\right)}^{2}+{\left(y_{d,i}-y_{P,i}\right)}^{2}}, \end{array}$$

which has been used as a performance indicator in some previously published papers. *N* is the number of points on the prescribed or target curve. From the results, it is seen that the proposed path repairing technique is far superior to the exterior penalty technique used in the previous studies [1–5, 7, 8, 10–13]. Based on the mean values of the objective function, all seven optimizer performances are greatly improved when implementing the path repairing algorithm as summarized in Table 3. All the optimizers can find the feasible solutions for all 30 runs. The optimizer that gives the best results is TLBO for both the mean value (*f*_obj_=0.3705) and the best result (*f*_obj_=0.0010). The second and third best optimizers are ABC and GWO, respectively. The worst performer in this case, according to Mean, is SCA. In Table 2, only ABC can search for feasible solutions for all 30 runs. Based on the mean objective values, the performance of all optimizers deteriorates compared to the results from using PRA in Table 1. The best in cases of using the exterior penalty function technique is ABC, while the second best is GWO as it can search for feasible solutions for 29 from 30 runs with lower mean objective value compared to JADE. Figures 2 and 3 show the path traced by the coupler point of the best solution and the kinematic diagram of best linkage, respectively. It is found that TLBO with the novel design repairing technique gives the best result (Error = 0.0122) better than previous work [5, 14] (Error = 0.1227 (DE), and 0.0166 (DE), respectively). This design result cannot be compared with the previous work [15] due to the number of evaluation of the present work that is lower than ten times when compared with the reference. The best actual path as shown in Figure 3 is closer to the target path than in previous work [5, 14] and comparable with [15]. The four-bar linkage obtained from the best solution as shown in Figure 3 can completely rotate in an ascending direction.

**Figure 4:**
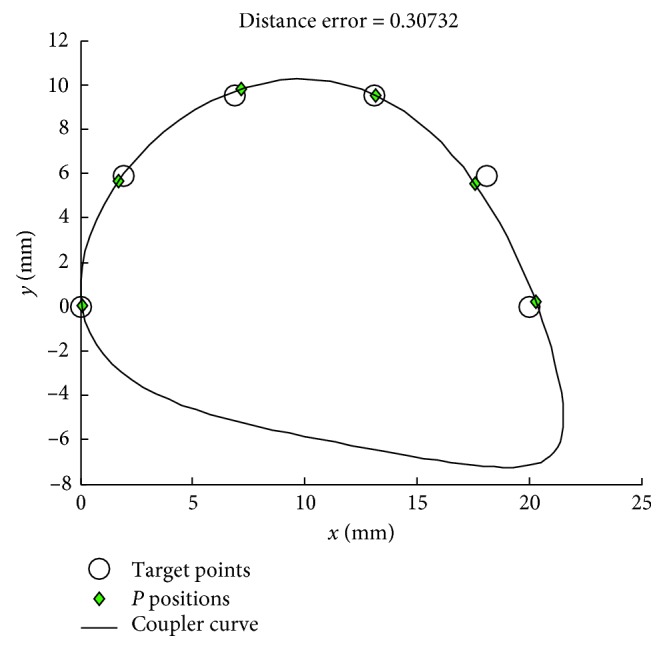
Best coupler curve obtained in Case 2.

**Figure 5:**
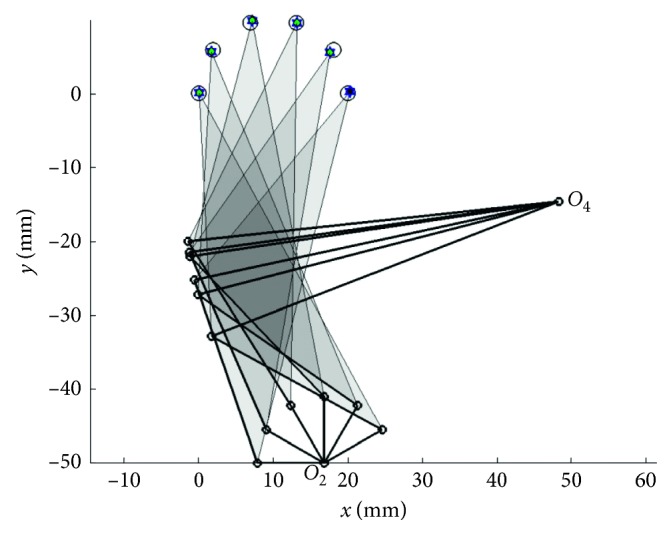
Best mechanism obtained in Case 2.

For Case 2 with the number of target points at 6, the results obtained from using the various MHs with PRA and the penalty function technique are shown in Tables 4 and 5, respectively. The coupler curves and the best linkages obtained from TLBO are shown in Figures 4 and 5, respectively. In this case, all MHs are significantly improved by using PRA based on the mean objective function values. They can find feasible solutions for all runs with the given predefined number of function evaluations. Note that PBIL using PRA has higher mean value compared to that using the penalty function technique, but this is computed from only 21 successful runs when using the penalty function technique. Based on using PRA in Table 4, the best method is TLBO while the second and third best algorithms are JADE and PBIL, respectively. The worst optimizer is SCA according to the mean value. In Table 5, based on using the penalty function technique, the top three performers are JADE, GWO, and ABC in that order. In this design case, TLBO produces the best solutions for both constraint handling techniques. For this case, it is proved that using the second repairing technique (Algorithm 2) is better than the penalty function technique. The best optimizer is TLBO as with the first case which gives the best error result as 0.3073, while the results from previous work [5, 13, 14] are error = 2.3496 (DE), 2.5998 (ICA), and 1.6063 (JADE), respectively. The best actual path as shown in Figure 4 is closer with the target path than the previous work [5, 13, 14]. The four-bar linkage obtained from the best solution as shown in Figure 5 can completely rotate in an ascending direction in accordance with the prescribed timing and the circular target path.

**Figure 6:**
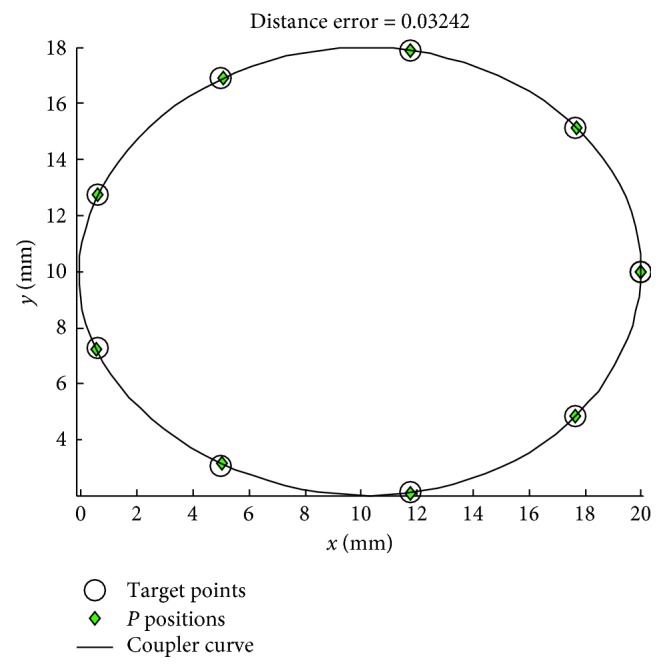
Best coupler curve obtained in Case 3.

**Figure 7:**
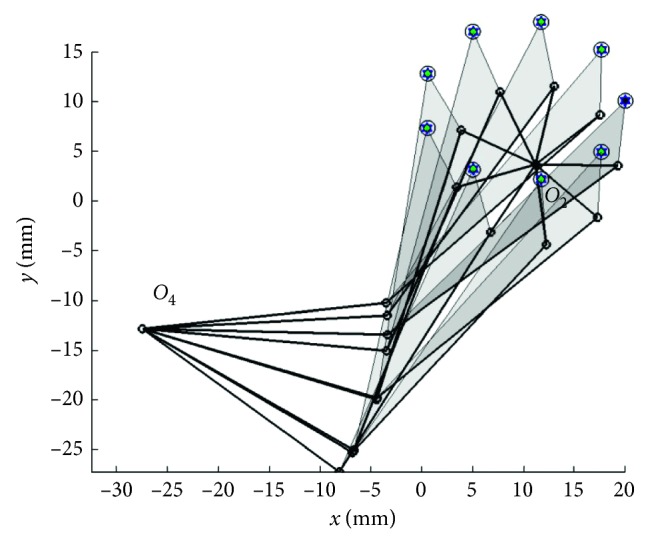
Best mechanism obtained in Case 3.

In Case 3 with the prescribed curve with 10 points, the results are obtained by those algorithms with PRA, and the penalty function techniques are shown in Tables 6 and 7, respectively. The coupler curve and the best linkage are shown in Figures 6 and 7, respectively. From the results, it is found that TLBO gives the best results for both the mean objective function value and the best result when using PRA. Similarly to the previous two cases, all MHs are greatly improved when using PRA. All the methods can find feasible solutions for all runs with using PRA. The top three performers in cases of using PRA are TLBO, ABC, and JADE in that order while the worst method is SCA based on the mean objective values. In the results of using penalty function technique, ACOR, and SCA cannot find a feasible solution. The best method when using penalty function technique is ABC while the performance of others cannot be evaluated as they can search for feasible solutions for a few optimization runs. The best result from TLBO gives an average distance error of 0.0324 while the previous work [5, 14] had error = 1.9523 (DE), and error = 0.1641 (DE), respectively. It means that the best actual path as shown in Figure 6 is closer with the target path than the previous work [5, 14]. The linkage obtained from the best solution as shown in Figure 7 can completely rotate in an ascending direction in accordance with the elliptic target path.

Table 3 gives a summary of the comparative performance of the various metaheuristics for solving the four-bar linkage path synthesis using the new constraint handling technique and the classical exterior penalty function technique. It shows that the results from using PRA are totally superior to those obtained from using the penalty function.

It is shown that TLBO with PRA is superior to the other MHs using the penalty technique. In this study, the Friedman test and the Tukey–Kramer test are used for comparing the results. Based on the Friedman test, TLBO ranks 1st whereas the second best is JADE at *p* value (0.02) < *α* (0.05) as shown in Table 8. It can be summarized that TLBO is the best performer for solving the four-bar path synthesis problems cases 1–3. Based on the Tukey–Kramer test, the mean column ranks of TLBO are significantly different from SCA.

The mean values obtained from using all optimizers of the test problems with PRA and the penalty function technique are shown in Table 3. In the statistical study, the results from ACOR and SCA are discarded because they cannot find any feasible solution in Case 3 (without using PRA). This table shows that MHs using the PRA approach give better mean than their counterparts that employ the penalty function technique for all test problems. The of average results of Friedman test are given in Table 9 which shows that MHs with the PRA technique significantly outperforms those using the penalty function technique at *p* value (0.00016) < *α* (0.05).

**Figure 8:**
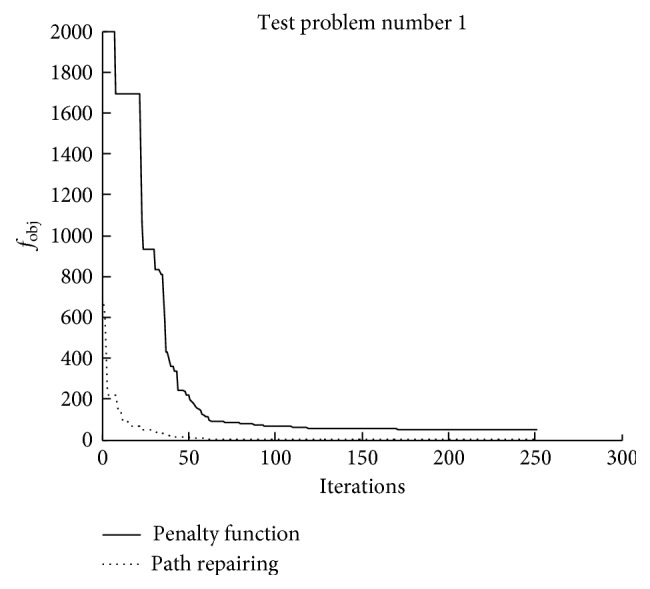
Search history of the best result obtained in Case 1.

**Figure 9:**
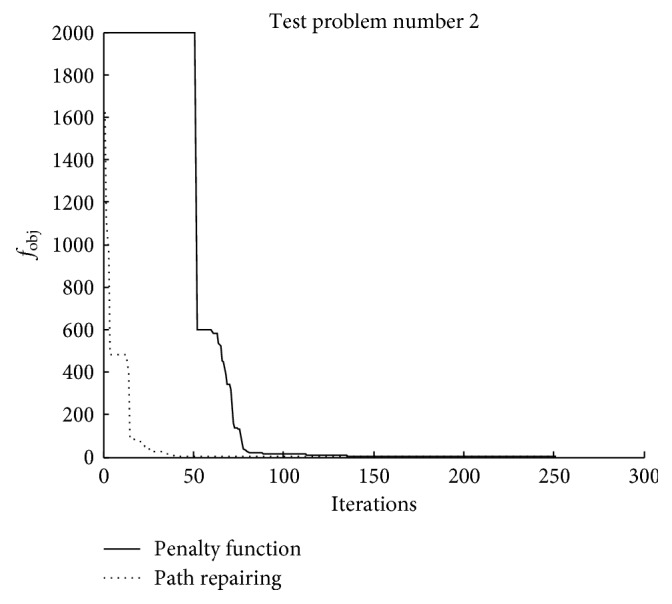
Search history of the best result obtained in Case 2.

**Figure 10:**
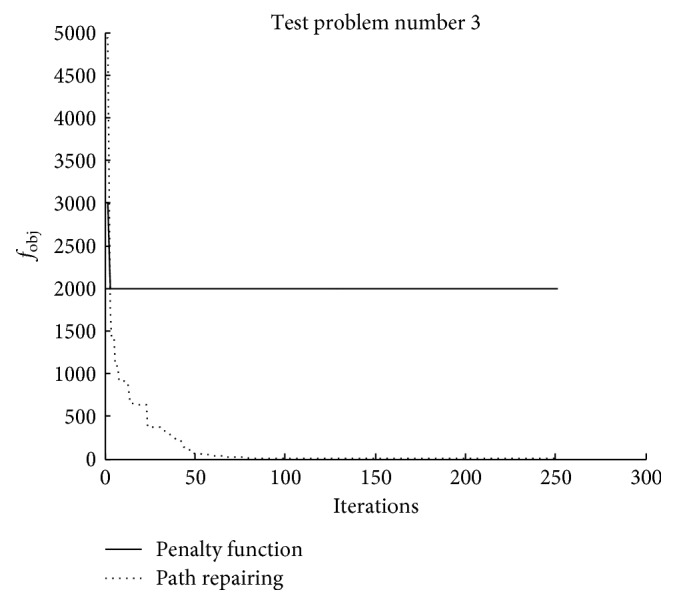
Search history of the best result obtained in Case 3.

Figures 8–10 illustrate the search histories of the best runs of TLBO for Case 1, Case 2, and Case 3, respectively. The search history compares the best runs obtained from using the penalty function technique and the path repairing algorithm. It can be seen that even though TLBO using PRA started with the worse solution (Case 3), the path repairing technique still guided the optimizer to converge considerably faster than when using the penalty function technique. All three figures show the superiority of the proposed path repairing technique for four-bar linkage path synthesis.

## 6. Discussion

**Figure 11:**
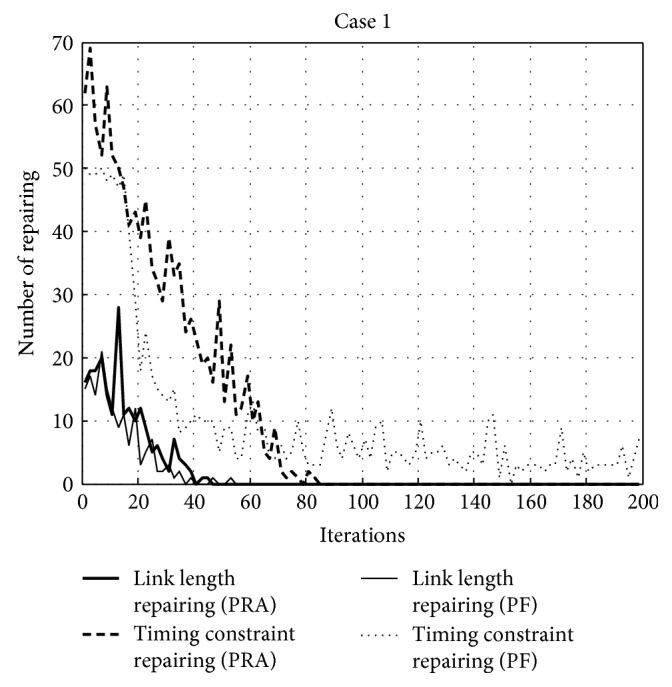
Repairing and penalty function histories of Case 1 for 200 iterations.

Further discussion is provided in order to investigate the behavior of the proposed constraint handling technique PRA and why it is efficient when used with TLBO.  Figure 11 shows the search history of the best runs of TLBO for the case 1 problem using PRA and the penalty function (PF) technique. The figure displays the number of iterations versus the number of infeasible solutions for both runs. The illustration also separates the number of infeasible solutions due to the timing constraint and those caused by the link length constraints. It can be seen that, with the use of PRA, infeasible solutions due to the link length constraints vanished after approximately 50 iterations. The same conclusion is applied in cases of using PF. By using PRA, the numbers of infeasible solutions due to the timing constraint disappear after around 85 iterations while the number of infeasible solutions due to the timing constraint when using PF cannot be suppressed throughout the search process. From this particular comparison, it is shown that the proposed PRA is efficient for dealing with both link length and timing constraints while the penalty function technique failed to solve the timing constraint problem.

**Figure 12:**
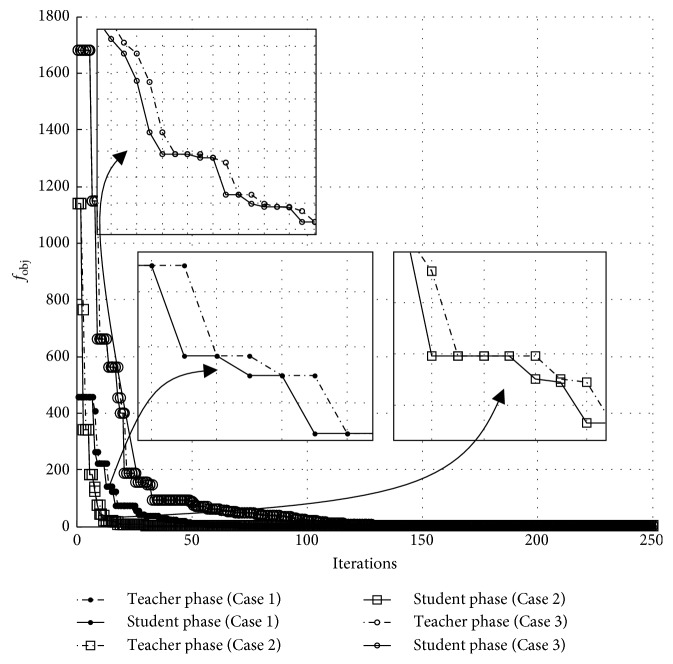
Teacher phase and student phase function evaluation history of Cases 1–3 for 500 iterations.

Figure 12 shows the search history (objective function versus iterations) of the best runs of the three design cases from using TLBO in combination with PRA. In this figure, the best objective function values obtained from the teaching and learning phases of TLBO are plotted separately. It can be seen from all three path synthesis problems that the best results produced by the learning phase are equal to or better than those obtained from the teaching phase. This implies that the reproduction using the learning operator of TLBO works well with the proposed PRA. In TLBO, the teaching phase is used for exploitation while the learning phase emphasizes more on exploration. That means the proposed technique tends to be efficient with an exploration-based reproduction operator. As a result, further development of TLBO for path synthesis of a four-bar linkage can adapt from the original version by adding a self-adaptive strategy for population sizing.

## 7. Conclusions

This paper presents a technique to find the optimum parameters of a four-bar linkage for path synthesis using metaheuristics including ABC, JADE, PBIL, TLBO, ACOR, GWO, and SCA. The new technique, called a path repairing technique, is proposed to handle the synthesis constraints effectively. The comparative results of the three case studies show that the new path repairing technique is superior to the penalty function technique traditionally used in four-bar linkage path syntheses in the previous studies. The comparative performance of the metaheuristics shows that the TLBO with PRA is the most efficient method based on both convergence rate and consistency. The results in this work can consider as the baseline for developing an efficient optimizer for four-bar linkage path syntheses in the future. Any optimizer should be tested by running it many times to solve the synthesis problems, and the mean value of an objective function should be used as a performance indicator.

## Figures and Tables

**Algorithm 1:**
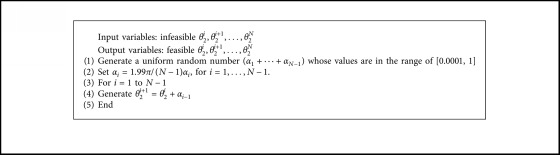
A repairing technique for the prescribed timing constraint.

**Algorithm 2:**
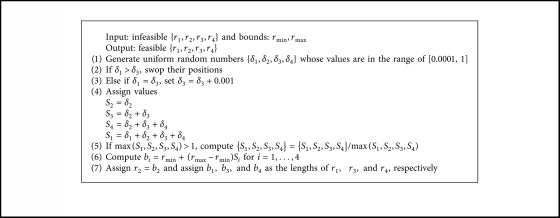
Repairing Grashof criterion constraint.

**Table 1:** Comparative results for Case 1 with a novel path repairing technique.

Case 1: path generation without prescribed timing							
Parameters	ABC	JADE	PBIL	TLBO	ACOR	GWO	SCA
*r*_1_	39.7861	52.4762	53.7995	57.9042	59.4760	33.4305	28.3704
*r*_2_	10.1505	14.3110	9.4303	8.5359	9.1050	9.8739	5.8563
*r*_3_	50.4447	48.0989	44.1070	20.5846	51.8860	38.5048	60.0000
*r*_4_	35.2568	47.5363	46.5599	55.5223	20.4784	53.3193	38.7556
*r*_*cx*_	45.1511	30.2155	47.8722	5.0306	−6.9474	37.5632	60.0000
*r*_*cy*_	7.2885	−14.8707	32.8201	35.6648	−21.9732	−10.3487	−60.0000
*x*_2_	50.7782	11.7094	60.0000	47.0842	−8.8106	−5.2992	−60.0000
*y*_2_	−0.9366	−3.1269	−8.8920	12.5554	28.8944	59.6847	60.0000
*θ*_0_	82.2709	51.1387	45.4292	355.3772	96.7425	234.8353	0.0000
*θ*_2_^1^	288.6774	313.0937	352.6949	9.4470	276.1848	334.3267	0.0000
*θ*_2_^2^	18.7094	338.5807	24.3156	23.8648	298.9037	5.9786	71.2728
*θ*_2_^3^	39.4220	359.8582	29.9612	37.4934	337.6217	31.4398	86.9490
*θ*_2_^4^	57.0069	18.5113	56.1299	51.6572	358.4935	61.3596	103.9052
*θ*_2_^5^	75.0134	44.8517	73.4859	67.9991	33.9200	90.2248	130.0967
*θ*_2_^6^	103.2820	85.9854	136.6204	91.0354	57.5750	139.0187	166.4231
Min	0.6374	1.8255	6.7539	**0.0010**	49.1961	0.3271	42.5282
Mean	4.1297	7.5933	20.0681	**0.3705**	73.1582	8.5501	144.5432
Max	11.9015	17.0022	47.5797	2.7424	101.7039	94.4204	391.5969
Std	2.7684	3.4092	10.1640	0.6667	16.1132	17.0012	94.5604
Error	0.2380	0.4502	0.8232	0.0122	2.6807	0.2107	2.6387
Success	30	30	30	30	30	30	30

Success = number of successful runs.

**Table 2:** Comparative results for Case 1 with a penalty technique.

Case 1: path generation without prescribed timing							
Parameters	ABC	JADE	PBIL	TLBO	ACOR	GWO	SCA
*r*_1_	38.2010	31.9116	34.6475	59.9108	22.0645	43.6675	60.0000
*r*_2_	8.6064	7.4478	6.4582	29.9172	5.0000	5.3499	5.0000
*r*_3_	22.4589	43.6552	21.6196	59.9967	60.0000	26.5380	47.3444
*r*_4_	33.2769	31.8355	23.7955	54.2963	60.0000	22.5663	60.0000
*r*_*cx*_	20.0224	9.4994	30.1437	59.7723	4.2836	−17.2375	60.0000
*r*_*cy*_	24.8915	25.3461	−25.6646	11.4222	11.0367	43.5804	−60.0000
*x*_2_	−5.1270	−0.4792	51.7915	41.5439	32.8024	59.9713	−60.0000
*y*_2_	52.6358	38.6191	54.5888	−2.4092	29.3709	10.5553	−0.6986
*θ*_0_	212.3407	224.9538	211.7426	47.8784	15.3431	16.7149	0.0000
*θ*_2_^1^	307.2217	241.9357	211.4543	304.0545	159.2096	342.4006	21.6624
*θ*_2_^2^	357.3637	226.6521	243.5720	347.0862	246.5489	15.3455	201.7648
*θ*_2_^3^	26.8761	204.4762	287.1713	359.9988	253.5892	37.5406	75.3665
*θ*_2_^4^	52.9043	174.1217	311.7283	9.0531	299.5365	60.6278	3.1728
*θ*_2_^5^	80.7316	141.3107	333.2289	16.0694	0.0000	91.7984	0.0000
*θ*_2_^6^	110.8412	131.9133	58.1996	22.0740	111.4815	134.0130	360.0000
Min	0.9797	23.1751	23.1950	0.2399	523.5085	0.8853	358.3843
Mean	23.4148	297.8795	133.3815	175.9177	1005.078	147.9292	452.8526
Max	78.2443	1151.615	247.9302	1106.170	1541.802	399.6278	562.5967
Std	18.2255	242.1490	74.2606	226.5618	236.8720	120.4374	102.9596
Error	0.3545	1.6529	1.7811	0.1808	7.9631	0.3532	5.9932
Success	30	29	6	26	22	29	3

Success = number of successful runs.

**Table 3:** Comparative Min and Mean for Cases 1–3 with a novel path repairing technique and a penalty technique.

Case number	With	Parameters	ABC	JADE	PBIL	TLBO	ACOR	GWO	SCA
1	PRA	Min	0.6374	1.8255	6.7539	**0.0010**	49.1961	0.3271	42.5282
		Mean	4.1297	7.5933	20.0681	**0.3705**	73.1582	8.5501	144.5432
		Success	30	30	30	30	30	30	30
	Penalty technique	Min	0.9797	23.1751	23.1950	0.2399	523.5085	0.8853	358.3843
		Mean	23.4148	297.8795	133.3815	175.9177	1005.078	147.9292	452.8526
		Success	30	29	6	26	22	29	3

2	PRA	Min	7.3696	0.7614	2.7790	**0.7614**	3.6197	1.0934	22.7680
		Mean	31.4436	13.3175	24.3873	**9.2918**	31.1379	27.5451	147.5295
		Success	30	30	30	30	30	30	30
	Penalty technique	Min	6.1512	8.5978	2.2458	0.7614	24.4416	2.6452	43.5787
		Mean	58.0879	35.9131	20.6274	45.8361	98.6229	39.3821	340.6984
		Success	30	30	21	29	30	30	30

3	PRA	Min	1.1331	2.2551	41.9444	**0.0192**	156.6075	26.8977	306.5633
		Mean	7.7983	9.2387	116.5084	**2.5706**	310.5264	70.7824	568.2001
		Success	30	30	30	30	30	30	30
	Penalty technique	Min	1.0714	1212.697	42.6040	352.3451	0.0000	251.2720	0.0000
		Mean	38.2367	1212.697	402.5957	576.6454	0.0000	493.2413	0.0000
		Success	29	1	4	10	0	15	0

**Table 4:** Comparative results of Case 2 with a novel path repairing technique.

Case 2: path generation with prescribed timing							
Parameters	ABC	JADE	PBIL	TLBO	ACOR	GWO	SCA
*r*_1_	50.0000	47.3318	37.3034	47.3319	47.0787	48.7592	23.7320
*r*_2_	8.7953	8.9594	9.7011	8.9594	9.5109	8.8102	6.4302
*r*_3_	17.5326	26.1415	27.5790	26.1415	23.8293	23.8043	43.5310
*r*_4_	50.0000	50.0000	40.1382	50.0000	44.9649	45.6184	37.4296
*r*_*cx*_	35.9767	43.5295	38.4166	43.5296	47.1950	49.8852	15.6308
*r*_*cy*_	−7.7228	−27.9916	−31.4443	−27.9914	−14.3055	−13.8744	−50.0000
*x*_2_	16.0681	16.8224	16.2277	16.8224	17.8426	16.9234	14.9035
*y*_2_	−34.3113	−50.0000	−48.1851	−50.0000	−47.7051	−49.9768	−50.0000
*θ*_0_	31.3196	48.3625	59.5519	48.3624	45.2184	44.2373	107.4695
Min	7.3696	0.7614	2.7790	0.7614	3.6197	1.0934	22.7680
Mean	31.4436	13.3175	24.3873	9.2918	31.1379	27.5451	147.5295
Max	52.3994	26.1423	92.0479	17.8459	107.1751	192.4375	660.2919
Std	13.9207	7.9546	18.4786	8.1308	23.2712	36.4676	141.0674
Error	1.0356	0.3073	0.6318	0.3073	0.6749	0.3723	1.6759
Success	30	30	30	30	30	30	30

Success = number of successful runs.

**Table 5:** Comparative result of Case 2 with a penalty technique.

Case 2: path generation with prescribed timing							
Parameters	ABC	JADE	PBIL	TLBO	ACOR	GWO	SCA
*r*_1_	40.7603	32.5637	50.0000	47.3284	47.6986	49.0512	25.7137
*r*_2_	5.8249	7.2738	8.9238	8.9594	6.4661	7.9356	5.0000
*r*_3_	9.9768	22.0128	23.9506	26.1434	21.0563	21.2936	25.7783
*r*_4_	38.6534	31.9534	46.8513	50.0000	48.2501	41.6373	38.5477
*r*_*cx*_	28.0556	42.1632	48.6935	43.5248	46.0280	50.0000	15.3979
*r*_*cy*_	−5.7200	−28.2724	−16.8126	−27.9988	−23.7354	3.4330	−50.0000
*x*_2_	12.9887	12.8441	17.1186	16.8220	12.5560	16.2388	12.3447
*y*_2_	−24.4168	−48.8226	−49.3931	−50.0000	−50.0000	−47.9755	−50.0000
*θ*_0_	34.5068	57.2910	46.9004	48.3661	41.6402	36.6935	67.0180
Min	6.1512	8.5978	2.2458	0.7614	24.4416	2.6452	43.5787
Mean	58.0879	35.9131	20.6274	45.8361	98.6229	39.3821	340.6984
Max	168.5279	97.6451	86.4201	609.1712	173.0658	108.0179	1064.047
Std	44.3671	17.0311	20.9987	139.0780	39.9827	32.7100	265.7480
Error	0.8997	1.0665	0.5467	0.3073	1.9211	0.6451	2.5063
Success	30	30	21	29	30	30	30

Success = number of successful runs.

**Table 6:** Comparative results of Case 3 with a novel path repairing technique.

Case 3: path generation without prescribed timing							
Parameters	ABC	JADE	PBIL	TLBO	ACOR	GWO	SCA
*r*_1_	80.0000	79.9162	69.0571	42.0053	66.5453	43.5326	80.0000
*r*_2_	8.1263	9.7027	10.0219	8.0876	10.6498	19.6630	5.1563
*r*_3_	80.0000	79.6300	80.0000	28.2660	65.3047	66.2180	64.8837
*r*_4_	51.8439	22.1050	66.3977	24.1099	62.9137	42.3820	61.7959
*r*_*cx*_	−19.3276	13.4224	−6.0994	−4.4860	6.0790	25.1410	−40.4117
*r*_*cy*_	−11.0023	−10.1904	−27.8102	−4.7935	−6.9436	−25.6124	−80.0000
*x*_2_	−1.2988	25.0942	−13.1694	11.1765	2.5885	−16.7352	25.4964
*y*_2_	29.1004	3.1796	26.4926	3.5870	7.9850	8.3961	−80.0000
*θ*_0_	53.9222	176.9942	12.1922	203.0714	0.0000	10.8144	162.9594
*θ*_2_^1^	349.6393	0.0377	8.8375	359.1656	52.8186	75.4675	47.9605
*θ*_2_^2^	35.6886	31.3880	45.3792	38.0948	76.7817	88.6364	77.3485
*θ*_2_^3^	77.1187	73.5207	79.6200	76.7126	111.7879	107.0112	78.7932
*θ*_2_^4^	115.8808	117.4221	91.7790	115.5382	139.0174	129.7889	102.9839
*θ*_2_^5^	153.8239	160.1615	140.7557	154.7689	168.2661	155.2651	132.9758
*θ*_2_^6^	193.8898	201.3488	196.8064	196.1914	196.3339	189.1377	158.4142
*θ*_2_^7^	234.8222	238.3213	225.1631	236.9290	236.0848	228.5280	178.2059
*θ*_2_^8^	275.8371	275.8487	272.5570	277.6224	269.5633	255.6323	216.7998
*θ*_2_^9^	311.5613	312.9753	298.1521	319.1629	298.2293	276.9471	227.5118
*θ*_2_^10^	348.9137	355.2115	360.0000	359.1397	322.3910	290.1490	239.6187
Min	1.1331	2.2551	41.9444	0.0192	156.6075	26.8977	306.5633
Mean	7.7983	9.2387	116.5084	2.5706	310.5264	70.7824	568.2001
Max	30.3123	25.7054	306.4299	28.5182	551.2737	234.2163	1144.216
Std	7.1036	4.9496	62.3238	6.4004	83.5219	43.5185	153.6016
Error	0.2925	0.4315	1.8480	0.0324	3.5846	1.4398	5.2041
Success	30	30	30	30	30	30	30

Success = number of successful runs.

**Table 7:** Comparative results of Case 3 with a penalty technique.

Case 3: path generation without prescribed timing							
Parameters	ABC	JADE	PBIL	TLBO	ACOR	GWO	SCA
*r*_1_	68.1732	19.9901	40.6029	17.7554	0.0000	59.7863	0.0000
*r*_2_	9.4618	5.8357	8.1608	5.0231	0.0000	5.0000	0.0000
*r*_3_	80.0000	43.1677	50.0664	32.9105	0.0000	74.9475	0.0000
*r*_4_	80.0000	50.3385	34.8760	31.2567	0.0000	30.6040	0.0000
*r*_*cx*_	−1.1813	18.7411	−0.3738	5.8641	0.0000	15.4730	0.0000
*r*_*cy*_	−13.1623	−25.0130	26.8058	24.6039	0.0000	80.0000	0.0000
*x*_2_	−1.1627	41.0708	26.7918	10.3944	0.0000	30.7488	0.0000
*y*_2_	16.3687	12.8362	30.3681	−16.0968	0.0000	−70.6740	0.0000
*θ*_0_	360.0000	134.8235	99.1181	309.4384	0.0000	4.4956	0.0000
*θ*_2_^1^	0.0000	172.6179	32.5643	351.2755	0.0000	360.0000	0.0000
*θ*_2_^2^	37.4238	325.2499	72.4952	52.7076	0.0000	34.6517	0.0000
*θ*_2_^3^	75.2680	337.0868	88.5868	94.6252	0.0000	102.8443	0.0000
*θ*_2_^4^	114.8952	20.4030	112.0993	143.0734	0.0000	135.9833	0.0000
*θ*_2_^5^	152.8726	182.4764	173.3556	190.6071	0.0000	164.5501	0.0000
*θ*_2_^6^	191.5270	331.0880	201.7909	317.9020	0.0000	186.7582	0.0000
*θ*_2_^7^	232.1421	82.2786	258.3224	129.4480	0.0000	188.6709	0.0000
*θ*_2_^8^	274.8734	134.4962	280.6872	300.9699	0.0000	8.0308	0.0000
*θ*_2_^9^	314.3132	241.5786	318.6172	335.0357	0.0000	19.9826	0.0000
*θ*_2_^10^	0.0000	303.1435	43.3601	357.2376	0.0000	51.8922	0.0000
Min	1.0714	1212.697	42.6040	352.3451	0.0000	251.2720	0.0000
Mean	38.2367	1212.697	402.5957	576.6454	0.0000	493.2413	0.0000
Max	110.6796	1212.697	781.4347	990.3743	0.0000	1901.836	0.0000
Std	25.2906	0.0000	398.2755	210.1756	0.0000	402.5266	0.0000
Error	0.3074	10.1061	1.9909	4.9788	0.0000	4.6902	0.0000
Success	29	1	4	10	0	15	0

Success = number of successful runs.

**Table 8:** Average ranking and *p* value of performance index of MHs achieved by Friedman test.

Average ranking of each algorithm Friedman							*p* value
ABC	JADE	PBIL	TLBO	ACOR	GWO	SCA	
3.3333 (3)	2.6667 (2)	4.3333 (5)	1 (1)	5.6667 (6)	4 (4)	7 (7)	0.02

**Table 9:** Performance comparison of each case study with and without a novel path repairing technique for all algorithms.

*p* value	Average ranking of each technique Friedman	
	With PRA	Without PRA
0.00016	1.0556 (1)	1.9444 (2)

## Data Availability

The data used to support the findings of this study are included within the article.
